# A Protoplast System for CRISPR-Cas Ribonucleoprotein Delivery in *Pinus taeda* and *Abies fraseri*

**DOI:** 10.3390/plants14070996

**Published:** 2025-03-22

**Authors:** Barbara M. Marques, Daniel B. Sulis, Bethany Suarez, Chenmin Yang, Carlos Cofre-Vega, Robert D. Thomas, Justin G. A. Whitehill, Ross W. Whetten, Rodolphe Barrangou, Jack P. Wang

**Affiliations:** 1 Forest Biotechnology Group, North Carolina State University, Raleigh, NC 27695, USA; bmachad@ncsu.edu (B.M.M.); dbsulis@ncsu.edu (D.B.S.); bkstepps@ad.unc.edu (B.S.); cyang5@ncsu.edu (C.Y.); cacofre@ncsu.edu (C.C.-V.); 2 TreeCo, Raleigh, NC 27606, USA; rbarran@ncsu.edu; 3 Department of Forestry and Environmental Resources, North Carolina State University, Raleigh, NC 27695, USA; jwhiteh2@ncsu.edu (J.G.A.W.); rosswhet@ncsu.edu (R.W.W.); 4 Biotechnology Teaching Group, North Carolina State University, Raleigh, NC 27695, USA; rdthoma4@ncsu.edu; 5 Christmas Tree Genetic Program, North Carolina State University, Raleigh, NC 27695, USA; 6 Department of Food, Bioprocessing and Nutrition Sciences, North Carolina State University, Raleigh, NC 27695, USA

**Keywords:** CRISPR, genome editing, protoplasts, conifers

## Abstract

Climate change profoundly impacts the health, productivity, and resilience of forest ecosystems and threatens the sustainability of forest products and wood-based industries. Innovations to enhance tree growth, development, and adaptation offer unprecedented opportunities to strengthen ecosystem resilience and mitigate the effects of climate change. Here, we established a method for protoplast isolation, purification, and CRISPR-Cas ribonucleoprotein (RNP) delivery in *Pinus taeda* and *Abies fraseri* as a step towards accelerating the genetic improvement of these coniferous tree species. In this system, purified protoplasts could be isolated from somatic embryos with up to 2 × 10^6^ protoplasts/g of tissue and transfected with proteins and nucleotides, achieving delivery efficiencies up to 13.5%. The delivery of functional RNPs targeting *phenylalanine ammonia lyase* in *P. taeda* and *phytoene desaturase* in *A. fraseri* yielded gene editing efficiencies that reached 2.1% and 0.3%, respectively. This demonstration of RNP delivery for DNA-free genome editing in the protoplasts of *P. taeda* and *A. fraseri* illustrates the potential of CRISPR-Cas to enhance the traits of value in ecologically and economically important tree species. The editing system provides a foundation for future efforts to regenerate genome-edited forest trees to improve ecosystem health and natural resource sustainability.

## 1. Introduction

Forests occupy more than 31% of Earth’s terrestrial area and are the major determinants of soil composition, biodiversity, atmospheric carbon concentrations, and microclimate [1,2]. In addition, forests are critical for the economy of many countries as a source of wood for construction lumber, as well as pulp and paper [3]. However, climate change has severely threatened forestry. Increasing temperatures, CO_2_ concentration, and changes in precipitation patterns will likely reduce forest productivity, affecting timber supply. The pulp and paper manufacturing sectors are considered one of the main sources of greenhouse gas release. In 2021, it was estimated that this industry emitted 24.8 million metric tons of CO_2_ in the US [4]. The significant impact of pulp and paper production on the environment is associated with the complexity of the lignocellulosic biomass. Due to its physicochemical properties, lignin is a barrier to the isolation of cellulosic fibers by chemical pulping. Wood feedstocks with less lignin or more-degradable lignin can reduce the carbon footprint in the chemical pulping process [5,6]. Genetically manipulated wood feedstocks with changes in lignin content and composition showed improvements in pulp yield and could mitigate the emission of CO_2_ resulting from pulping by up to 20% [6]. In the southeastern US, *Pinus taeda* (Loblolly pine) is the most prominent source of timber and kraft pulp, and its plantations correspond to the largest forest-tree-planted areas of the country [7]. The genetic manipulation of *P. taeda* wood composition could greatly contribute to more sustainable pulp and paper production.

Global climate change impacts also extend to native tree populations, increasing the risk of extinction for multiple forest species. Temperature warming facilitates the rapid spread of local and invasive pests to new vulnerable biomes, such as high-elevation forests, leading to drastic ecological consequences [8,9]. For example, *Abies fraseri* (Fraser fir) is the most popular Christmas tree species in the US and endemic to the high-elevation ecosystems of the Southern Appalachian Mountains [10]. Studies predict the native *A. fraseri* populations have a significant risk of extinction due to the increase in global temperatures. Also, *A. fraseri* survival is threatened due to its extreme susceptibility to insect and disease threats including Phytophthora root rot and the balsam woolly adelgid (BWA) [11,12,13]. The development of resistant *A. fraseri* genotypes through genetic manipulation could prevent further population decrease in this conifer species in the Southern Appalachian Mountains.

One of the primary bottlenecks in conifer biotechnology is the functional characterization of genes involved in key traits, such as wood formation and stress responses. This is critical to identify target genes and inform genome editing strategies. The exceptionally large genome sizes of conifers—ranging from 9 to 35 Gbp—and the abundance of large repetitive sequences, present substantial challenges for whole-genome sequencing and consequently gene discovery and validation [14,15]. In particular, non-model species such as *A. fraseri* remain largely unexplored at the functional genomics level. Developing efficient tools to characterize gene function is crucial for accelerating genetic improvement efforts. In *Populus* spp., for instance, protoplast-based systems have been successfully employed to investigate transcription factors regulating wood formation, underscoring the potential of protoplasts as a platform for functional genomics studies [16,17,18].

The use of CRISPR-Cas ribonucleoprotein (CRISPR-RNP) delivery presents a powerful and precise approach for gene editing in plant systems. This method enables rapid, transgene-free genetic modifications by directly introducing pre-assembled CRISPR-RNP complexes into protoplasts. PEG-mediated transfection of the CRISPR-RNPs has been successfully applied in various plant species, including wild tobacco (*Nicotiana attenuata*), lettuce (*Lactuca sativa*), and potato (*Solanum tuberosum*) [19,20]. While CRISPR-RNP delivery has gained traction in crop research, its application in forest trees remains largely unexplored. Notable exceptions include studies demonstrating CRISPR-driven mutations in rubber tree (*Hevea brasiliensis*) [21] and chestnut (*Castanea sativa*) [22]. Both studies targeted the mutation of a *phytoene desaturase* (*PDS*) gene. *PDS* is involved in the biosynthesis pathway of chlorophyll, carotenoids, and gibberellin [23]. Loss-of-function edits to *PDS* results in an albino phenotype, and it has been broadly employed for the validation of the CRISPR-Cas genome editing method across several plant species, including cabbage (*Brassica oleracea*), oilseed rape (*Brassica napus*), banana (*Musa* spp.), and rice (*Oryza sativa*) [24,25,26]. In conifers, however, research on CRISPR-Cas-based genome editing is still in its early stages, with reports limited to Larch (*Larix kaempferi*) [27] and *Pinus radiata* [28]. In addition, protoplast regeneration for both *A. fraseri* and *P. taeda* remain to be developed. Expanding CRISPR-RNP delivery to conifer species could provide a transformative tool for functional genomics and genetic improvement in these ecologically and economically vital trees.

In this study, we evaluated protoplast isolation and CRISPR-RNP delivery efficiency in *P. taeda* and *A. fraseri*. By systematically optimizing protoplast isolation and transfection procedures, we established conditions suitable for efficient CRISPR-RNP transfection and targeted gene knockout. CRISPR-induced mutations were detected in RNP-transfected protoplasts from both species, demonstrating the feasibility of this approach for genome editing in conifers. Given the ecological and economic significance of *P. taeda* and *A. fraseri*, establishing a reliable genome editing platform through CRISPR-RNP delivery in protoplasts could accelerate the development of climate-resilient forest trees and more sustainable bioproducts. Traits such as improved wood quality, enhanced disease resistance, and increased tolerance to abiotic stress could be efficiently engineered using this transgene-free system, ultimately contributing to the mitigation of climate change impacts on forestry [29,30].

## 2. Results

### 2.1. CRISPR-sgRNA Design for Knockout of the P. taeda PAL Gene

Improvements in wood quality can be achieved through the manipulation of the monolignol biosynthesis pathway and the consequent reduction in lignin content in trees [31]. The intricate nature of monolignol biosynthesis hinders the ability of traditional hypothesis-driven approaches to quantitatively predict the impact of gene perturbations within the pathway on wood fiber properties. To evaluate the most impactful gene families for targeting with CRISPR, we used our established predictive model for monolignol biosynthesis [31,32,33,34]. We simulated the impact of each monolignol gene family knockout in lignin content. The predictive model simulation has shown that the knockout of the *Phenylalanine ammonia-lyase* (*PAL*) gene family displays the most impactful lignin content reduction among the gene families tested (32%) (Figure S1). PAL is a family of enzymes that catalyzes the deamination of phenylalanine to cinnamic acid, controlling the biosynthesis of all phenylpropanoid compounds, including lignin [35,36,37]. Ongoing research focusing on optimizing lignin content through *PAL* manipulation, ensuring wood quality while considering the intended applications and overall tree physiology have been demonstrated in *Populus trichocarpa* [6,31]. Given the importance of this family, *PtPAL*, the only *PAL* gene member identified in *P. taeda*, was chosen as the target gene. To find potential target regions and design CRISPR-sgRNAs for the knockout of *PtPAL*, we first needed to confirm its gene sequence and identify potential allelic variations. Primers were designed for the amplification of the 5′ CDS portion of *PtPAL* based on its complete CDS deposited on Genbank (Accession Number: U39792.1). The amplicons were sequenced through Sanger and analyzed with alignment tools. The sequencing results revealed six SNPs in positions 21, 30, 292, 385, 429, and 532 bp compared to the reference sequence. In addition, a 26 bp gap was identified in one of the sequenced reads, possibly indicating the presence of more than one variant of *PtPAL* in the *P. taeda* genome (Figure S2A).

After the confirmation of the *PtPAL* CDS, we designed five sgRNA sequences to target the 5′ end of the gene CDS, to ensure the complete deactivation of *PtPAL* expression. Considering that different sgRNAs sequences have variable cleavage efficiencies, we selected sgRNAs to target five different positions of the *PtPAL* 5′ region (Figure S2B). The selected sgRNAs displayed the highest specificity and efficiency scores in silico according to prediction software analysis (Table 1). A high specificity minimizes off-target effects, reducing the risk of unintended mutations, while a high efficiency ensures effective cleavage at the target site, enhancing the likelihood of successful gene disruption [38]. In the CRISPOR prediction tool (https://crispor.gi.ucsc.edu/, access date 10 March 2025), two methods are employed to evaluate sgRNA efficiency: Doench’16, which better predicts the efficiency of sgRNAs expressed in vivo under the U6 promoter; and Moreno-Mateos, which more accurately predicts the efficiency of sgRNAs expressed in vitro with a T7 promoter. Also, all sgRNAs targeted conserved regions within both *PtPAL* allelic variants and its Genbank annotated CDS. To screen for sgRNAs that could drive the cleavage of the designed target sequences in vitro, we carried out CRISPR-Cas cleavage assays. The assays showed that all five sgRNAs efficiently cleaved *PtPAL* at the predicted positions (Figure S3A), confirming the specificity of the designed sgRNAs to their target sequences in vitro. The cleavage assays demonstrated that any of the five sgRNAs screened could potentially be utilized for the knockout of *PtPAL* in vivo through CRISPR-RNP protoplast transfection. Since many factors can affect genome editing efficiency in vivo, such as target accessibility, chromatin state, and DNA conformation, screening a set of validated sgRNA sequences is essential to achieving genome editing in vivo.

### 2.2. Initiation of Pinus taeda Somatic Embryogenic Tissue Cultures

Somatic embryogenesis (SE) is a widely established method for the clonal propagation of conifer tree species. In this process, the proliferation of zygotic embryos is induced in vitro, allowing for the rapid and scalable production of embryogenic tissue (ET) masses [39]. Due to its uniformity and totipotency, ET masses are a suitable tissue source for protoplast isolation and transfection. SE initiation for *P. taeda* was performed according to a protocol described by Pullman et al., 2018 [39]. A total of 2995 seeds harboring zygotic embryos ranked from developmental stages 2 to 4 [40] were disinfected and dissected. We extracted mega-gametophytes from 1537 seeds, and zygotic embryos from the other 1458 seeds. After 80 days of incubation in PTIM medium [39], we obtained 79 growing ET lines, which corresponded to 2.63% of the total number of seeds dissected. Initiation from megagametophytes displayed a recovery rate of 3.25% while 2% of the zygotic embryos produced ET. The wide range of ET lines initiated in this experiment provided us with a diversity of genotypes with varying potential for the development of protoplast transfection.

### 2.3. Overnight Cell Wall Digestion Improves Transfection Efficiency of P. taeda Protoplasts

**Figure 1:**
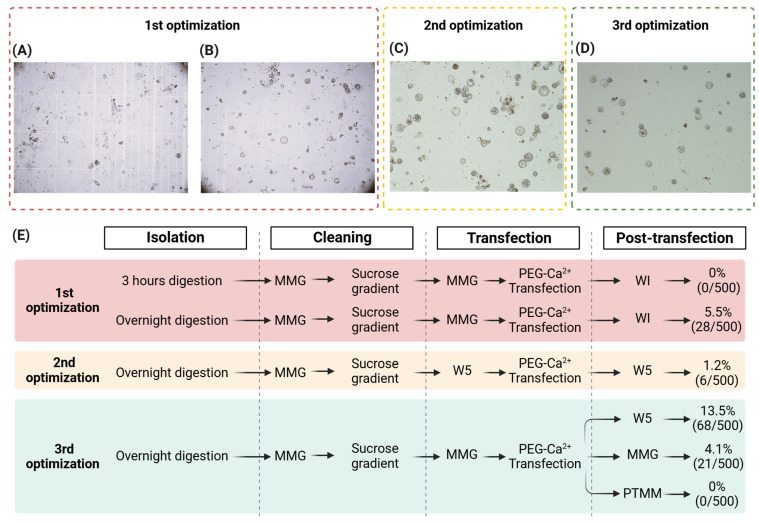
Establishment and optimization of the protocol for the efficient isolation and transfection of the *P. taeda* SE-originated protoplasts. In the first optimization step, *P. taeda* ET from line 21-103 was digested for protoplast isolation after 3 h (**A**) and overnight (**B**) incubation. After digestion, protoplasts were purified through a sucrose gradient and transfected with the pUC19-*CaMV35S*-*gfp* reporter plasmid. At the second optimization, protoplasts from the 21-330 SE line were isolated overnight (**C**), suspended in W5 buffer for pUC19-*CaMV35S*-*gfp* plasmid transfection, and incubated in W5 for 16 h. In the third optimization, overnight-isolated protoplasts (**D**) were transfected with reporter plasmid in MMG, and incubated in different post-transfection buffers, including W5. (**E**) Flow chart representation of the detailed procedures adopted for each optimization step. Transfection efficiencies resulting from each optimization were accessed through the transfection of the pUC19-*CaMV35S*-*gfp* reporter plasmid, where the GFP-positive cell rate was calculated by the proportion of green-fluorescent protoplasts under UV light within a total of 500 protoplasts.

For the initial isolation of the *P. taeda* SE-originated protoplasts, we adapted the procedures described for the isolation of *P. trichocarpa* xylem protoplasts [41], which demonstrated highly efficient protoplast isolation yields. The initial isolation performed under 3 h incubation in CWDS resulted in 2 × 10^5^ protoplasts/gram of ET and displayed a high amount of undigested tissue and debris. To increase the proportion of fully digested protoplasts, a protoplast purification step was performed using a sucrose gradient (see Section 4 Materials and Methods). Purified protoplasts were resuspended in MMG, transfected with a pUC19-*CaMV35S*-*gfp* reporter plasmid, washed, and incubated in WI solution. The transfection efficiency was assessed after 16 h, and no GFP-positive protoplasts were observed (Figure 1A). The abundance of cell debris in the protoplast sample was a clear indication that the cell wall digestion step required further optimization.

To address this issue, we searched for alternative cell wall digestion methods. In *Nicotiana benthamiana*, high yields of the viable protoplasts were obtained through overnight incubation in digestion solution supplemented with culture medium [42]. To test this method, we incubated *P. taeda* ET in CWDS2 (CWDS1 supplemented with PTMM) overnight and maintained the same steps for the transfection of isolated cells with the reporter plasmid. After digestion, the yield of SE-originated protoplasts increased slightly to 2.7 × 10^5^ protoplasts/gram of SE tissue, and the amount of undigested debris considerably decreased (Figure 1B). In addition, despite the extended cell wall digestion period, 87% of the protoplasts remained viable after 16 h of incubation (Figure S6). The transfection of pUC19-*CaMV35S*-*gfp* increased to 5.5%, in comparison with the previous experiment (Figure 1E). Overall, the overnight digestion method improved the isolation of viable protoplasts from the *P. taeda* SE masses and increased transfection efficiency. Even though clear improvements were obtained, we observed that the transfected protoplasts adopted a shriveled shape after 16 h, which was an indication of osmotic imbalance between protoplasts and the buffering conditions provided in WI. Therefore, further optimization of the transfection and post-transfection steps were still needed.

### 2.4. Transfection Buffer Conditions Affect Protoplast DNA-Uptake Efficiency

To investigate the shriveling condition and optimize the osmotic balance for the *P. taeda* SE protoplasts, we tested a range of buffering conditions during transfection, and post-transfection incubation periods. Due to the successful implementation of the protocol steps from Hsu et al., 2021 [42] in the previous protoplast isolation, we examined whether its transfection settings could also improve *P. taeda* protoplast DNA uptake and recovery. In this protocol, W5 buffer was employed as the main solution for all washing and incubation steps during protoplast isolation and transfection. To test this, we carried out an overnight digestion of the *P. taeda* SE masses in CWDS2. Isolated protoplasts were then purified through a sucrose gradient and resuspended with W5 buffer for transfection with pUC19-*CaMV35S*-*gfp*. After transfection, protoplasts were washed and incubated in W5, and the transfection efficiency was assessed after 16 h. The isolation yielded 3.26 × 10^5^ cells/gram of SE tissue. Most cells exhibited a fully rounded shape, as expected for healthy protoplasts, indicating that the W5 composition was ideal for the maintenance of osmotic balance in *P. taeda* protoplasts (Figure 1C). Despite the substantial improvement of the osmotic condition, transfection efficiency dropped to 1.2% (Figure 1E). Altogether, these results demonstrated that even though W5 provided osmotic stability, it negatively interfered with protoplast DNA uptake in the transfection step.

### 2.5. W5 Buffer Facilitates Post-Transfection Recovery of Protoplasts

The formulation of ideal conditions that enable protoplasts to undergo survival, recovery, and development after transfection is essential for the successful recovery and regeneration of CRISPR-RNP-edited protoplast samples. In the previous experiment, albeit with the increase in protoplast viability, the transfection efficiency was compromised with the employment of W5 buffer. At this point, the experiment displaying the highest transfection efficiency was performed with MMG as a transfection buffer. Because of that, we decided to test whether the use of MMG buffer as a pre-transfection resuspension buffer, in combination with W5 as the post-transfection incubation buffer, could rescue the transfection efficiency rates. Protoplasts were isolated following the previous procedures, purified and resuspended in MMG, and then transfected with reporter plasmid. After transfection, protoplasts were washed with W5, MMG, or PTMM media supplemented with 0.5 M mannitol, and incubated overnight in the same respective buffers. The observation of GFP fluorescence showed that the protoplasts incubated with W5 displayed a transfection efficiency of 13.5% (Figure 1D), the highest among all tested conditions, which were 4.1% (Figure S4A,B) and 0% for MMG and PTMM-mannitol (Figure S4C,D), respectively. The results demonstrate that the employment of MMG in the transfection, and subsequent post-transfection incubation in W5 considerably enhanced DNA uptake and viability of the *P. taeda* SE-originated protoplasts and provided favorable conditions for the transfection of CRISPR-RNP complexes and the recovery of CRISPR-edited protoplasts.

### 2.6. Genome Editing of PtPAL Through CRISPR-RNP Protoplast Transfection in P. taeda

**Figure 2:**
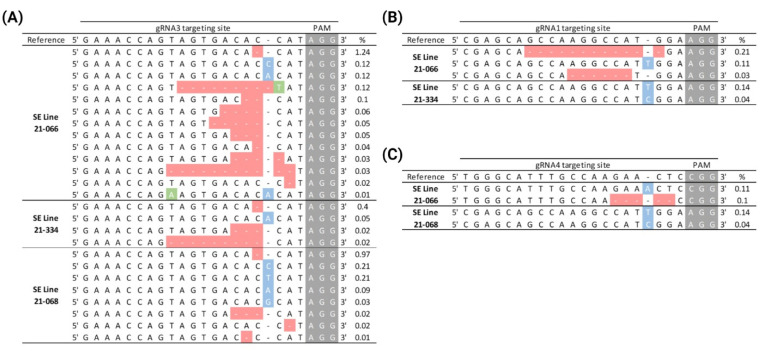
CRISPR-mediated *PtPAL* knockout through the RNP transfection of SE-originated protoplasts. *P. taeda* SE-isolated protoplasts from lines 22-066, 22-068, and 21-334 were isolated, and transfected with CRISPR-RNP complexes targeting different regions of the *PtPAL* gene sequence. Samples transfected with CRISPR-RNPs were analyzed through amplicon deep sequencing. CRISPR-associated *PtPAL* variants were observed for samples transfected with gRNA3 (**A**), gRNA1 (**B**), and gRNA4 (**C**) where base pairs highlighted in gray represent the PAM sites; in blue, insertions; in red, deletions; and in green, base pair exchanges.

The parameters established for the isolation and transfection of SE-originated *P. taeda* protoplasts (summarized in Figure 1E) led to an improved transfection efficiency from 0 to 13.5%, which enabled the employment of CRISPR-RNPs for gene editing. To target the knockout of *PtPAL*, we individually assembled the CRISPR-RNP complexes harboring the five gRNAs (sgRNA1-5) previously designed and validated in vitro for *PtPAL* cleavage. The assembled CRISPR-RNPs were then transfected into the *P. taeda* SE protoplasts following our isolation and transfection method. The transfection of CRISPR-RNPs was separately performed with protoplasts isolated from three distinct SE lines. After 72 h post-transfection, CRISPR-driven mutations of all five *PtPAL* target sites were assessed through amplicon sequencing (Genewiz, South Plainfield, NJ, USA). We observed that CRISPR-Cas editing efficiencies were considerably variable within the different assembled sgRNA and the SE lines transfected. CRISPR-editing events were observed for sgRNA1, sgRNA3, and sgRNA4. In contrast, sgRNAs 2 and 5 did not yield any editing event in the target positions of these sgRNAs on any of the SE lines. sgRNA3 was the most efficient among the sgRNAs used, and generated target editing events regardless of the SE protoplast source as well as displaying the highest percentage of INDELs among the SE lines tested (0.5 to 2.1%) (Figure 2A). For samples transfected with RNPs for sgRNA1 and sgRNA4, editing efficiency ranged from 0–0.35%

### 2.7. CRISPR-sgRNA Design for Knockout of the A. fraseri PDS Gene

To develop a protoplast system for CRISPR-driven genome editing in *A. fraseri*, we chose to target the *A. fraseri phytoene desaturase* (*AfPDS*) gene. *PDS* is involved in the biosynthesis pathway of chlorophyll, carotenoids, and gibberellin [23].

To date, whole-genome sequencing data for *A. fraseri*, or the closely related *Abies* species are not available. Therefore, to obtain the *AfPDS* sequence for CRISPR-sgRNA design, we evaluated a transcriptome assembly made using Trinity (Haas et al., 2013) [43] from existing transcriptome libraries (BioProject PRJNA357112, BioSample SAMN06133326, Sequence Read Archive, NCBI—Bethesda, MD, USA). The predicted *AfPDS* CDS contains 1752 bp and was used as a reference for the design of the primers and amplification of a 5′ end 215 bp exon fragment. The 215 bp amplicon was sequenced through Sanger and confirmed to be identical to the predicted *AfPDS* reference sequence.

After sequence validation, we designed sgRNAs for the *AfPDS* CRISPR-driven knockout. To do that, we used sgRNA predicting software to find sgRNA targeting positions within the 215 bp fragment previously validated. From this analysis, we selected four sgRNAs (sgRNA1-4) with the highest efficiency and outcome predicting scores to target *AfPDS* across the 5′ region (Table 1). To evaluate cleavage efficiency and target specificity of the designed gRNAs, we performed a CRISPR-Cas in vitro cleavage assay, using the *AfPDS* 215 bp fragment as a target template. All the sgRNAs were able to cleave *AfPDS* at the correspondent target positions (Figure S3B). This result suggests that despite the lack of a genome sequence for *A. fraseri*, we could identify functional sgRNAs that can be used to target *AfPDS* cleavage in vivo.

### 2.8. AfPDS Editing in CRISPR-RNP-Transfected A. fraseri Protoplasts

To formulate methods for the isolation and transfection of CRISPR-RNPs into *A. fraseri* protoplasts, we took advantage of an existing *A. fraseri* in vitro ET collection, developed by the Christmas Tree Genetics program at North Carolina State University. ET masses from two-week old in vitro cultures were embedded in CWDS1 and incubated for 3 h. Following digestion, we obtained an ET-originated protoplast yield ranging from 5 × 10^4^–3.8 × 10^5^ protoplast/gram of ET. The digested protoplasts were transfected with CRISPR-RNPs assembled with the four previously validated sgRNAs and the pUC19-*CaMV35S*-*gfp* reporter plasmid as a transfection control. After 16 h of post-transfection incubation, no GFP-fluorescent protoplasts were observed in the control sample, indicating very low plasmid delivery efficiency. Remarkably, we detected CRISPR-edited *AfPDS* variants in the protoplast sample transfected with sgRNA4 RNP, with an editing efficiency of 0.056% (Supplementary Figure S5). This suggested that RNP delivery may occur even if the fluorescence reporter for transfection efficiency is undetectable, and since the molecular weight of RNP (190–200 kDa) is substantially smaller than that of the pUC19-*CaMV35S*-*gfp* reporter plasmid (~2908 kDa), RNPs may be more efficiently delivered into the protoplasts.

**Figure 3:**
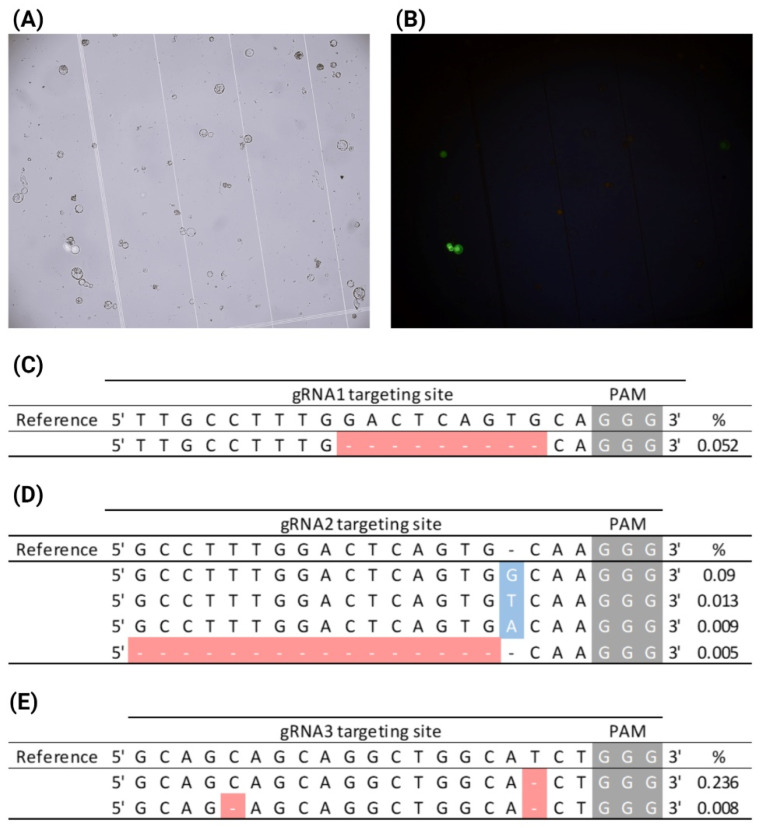
CRISPR-mediated *AfPDS* knockout through the RNP transfection of SE-originated protoplasts. The *A. fraseri* SE-isolated protoplasts from the 51-0025 line were isolated and transfected with CRISPR-RNP complexes targeting different regions of the *AfPDS* gene sequence. Additionally, protoplasts were transfected with the pUC19-GFP reporter plasmid. After 16 h of transfection, protoplasts were observed under bright field (**A**) and UV light (**B**) for the assessment of transfection efficiency. Samples transfected with CRISPR-RNPs were analyzed through amplicon deep sequencing. CRISPR-associated *AfPDS* variants were observed for samples transfected with sgRNA1 (**C**), sgRNA2 (**D**), and sgRNA3 (**E**), where base pairs highlighted in gray represent the PAM sites; in blue, insertions; and in red, deletions.

To improve the transfection efficiency and consequently increase CRISPR editing efficiency, we incorporated a sucrose gradient purification step for protoplast isolation. Similarly to *P. taeda*, we aimed to remove the cell debris and partially digested cells from the samples, thereby enriching the proportion of healthy protoplasts. To test the effect of protoplast purification, we maintained the procedures adopted in the first experiment for protoplast isolation and subsequently performed a sucrose gradient with the digested protoplasts. The purification process resulted in a ~50% reduction in protoplast yield but enhanced the homogeneity of the protoplasts with substantially fewer undigested cells and cell debris. Purified protoplasts were transfected with the CRISPR-RNPs assembled with the sgRNA collection and the reporter plasmid. We observed that 70% of the protoplasts remained viable after transfection, by accessing viability through FDA staining. After 16 h, green fluorescence was observed in 3.7% of the pUC19-*CaMV35S*-*gfp*-transfected protoplasts (Figure 3A,B). Successful CRISPR-Cas editing of *AfPDS* was observed for three of the four gRNAs tested (sgRNA1-3), with editing efficiencies ranging from 0.052 to 0.25% (Figure 3C–E). The enrichment of healthy cells in the sample through a protoplast purification method improved protoplast transfection, which led to the increased editing efficiency of *AfPDS*.

## 3. Discussion

In this study we demonstrated the isolation and transfection of SE-originated protoplasts from *P. taeda* and *A. fraseri* for a CRISPR-Cas delivery and genome editing proof of concept. For both species, we observed that cell wall digestion was a pivotal step for efficient isolation and further transfection. In addition, maintaining balanced osmotic pressure was crucial for preserving cytoplasmic integrity. Plant cell walls are effective in preventing the bursting or swelling of cells caused by external osmotic perturbations [44], and when removed, cells become sensitive to minor osmotic changes. Osmotic pressure stabilizers (e.g., glucose, mannitol, and salts) are critical to fine-tune the buffer osmotic pressure and reduce the effect of the cell wall’s absence during protoplast isolation and proliferation. Different plant tissues and cell types have varying osmotic needs, and therefore protoplast osmotic conditions usually require optimization according to the source tissue.

We showed that adjustments to the protoplast isolation and purification procedures can enhance transfection rates and increase editing efficiency through CRISPR. In *P. taeda*, we could increase the transfection efficiency from 0 to 5.5% by increasing the cell wall digestion time to an overnight incubation. In *A. fraseri*, an additional purification step through a sucrose gradient promoted the enrichment of fully digested protoplasts, increasing the transfection efficiency from 0 to 3.7% and *AfPDS* CRISPR-driven mutations from 0.056 to 0.25%. The purification step did not improve protoplast yield or viability. Overnight isolation has already been applied for CRISPR genome editing in other model plant species. Protoplasts were successfully isolated during overnight cell wall digestion in *Nicotiana benthamiana* [42].

We observed that the presence of magnesium chloride in the transfection reaction might facilitate protoplast DNA uptake in *P. taeda*, since it increased the transfection efficiency from 1.2 to 4.4%, compared with the W5 buffer. Studies in oil palm (*Elaeis guineensis*) protoplasts have shown that transfection efficiency proportionally increases with higher concentrations of magnesium chloride in the transfection reaction [45], which corroborates with the data we obtained for *P. taeda* protoplast transfection. The buffer composition is also critical for the recovery of transfected protoplasts. Our experiments demonstrated that the presence of a carbon source in the post-transfection incubation medium enriched the yield of GFP-positive cells in the sample, from 4.1 to 13.5% for samples incubated in MMG and W5, respectively.

Genome editing through the PEG-mediated delivery of CRISPR-RNP complexes has been established for many agricultural crop species, including wild tobacco (*Nicotiana attenuata*), lettuce (*Lactuca sativa*), and potato (*Solanum tuberosum*) [19,20]. However, reports on CRISPR-RNP delivery in trees are still limited and lag behind in adoption, and the delivery methods, as well as the editing efficiencies, vary broadly according to species. For fruit trees, PEG-mediated CRISPR-RNP transfection was explored in apple (*Malus domestica*), resulting in an editing efficiency of up to 6.9%, in banana (*Musa* spp.) with an editing efficiency up to 0.92%, and in grapevine (*Vitis vinifera*) with an efficiency up to 0.1% [26,46]. Regarding forestry species, editing with CRISPR-RNPs yielded up to 20.11% efficiency in rubber tree (*Hevea brasiliensis*) protoplasts and 21.4% in European chestnut (*Castanea sativa*) protoplasts [21,22].

Following the establishment of an isolation and transfection protocol for the delivery of CRISPR-RNP complexes into SE-originated protoplasts, we demonstrated CRISPR-Cas-driven genome editing in *P. taeda* and *A. fraseri*. We targeted the knockout of the *PtPAL* gene in *P. taeda* at five different gene positions and obtained editing efficiencies of up to 2.1%. The *PtPAL* sgRNA3 consistently displayed the highest editing efficiency among the sgRNAs tested. For *A. fraseri*, we obtained up to 0.25% efficiency in targeting the mutation of the *AfPDS* gene, where the *AfPDS* sgRNA3 displayed the highest efficiency. Several factors influence CRISPR-RNP-mediated editing in protoplasts, including target site accessibility, sgRNA secondary structure, and chromatin state. Additionally, increasing transfection efficiency is crucial for enhancing editing outcomes. The higher editing efficiencies observed in *P. taeda* compared to *A. fraseri* are likely due to a greater proportion of transformed protoplasts [47].

Our findings illustrated CRISPR-RNP delivery as a promising tool for the genome editing of conifer species. Further improvements in transfection and CRISPR-editing efficiencies may be explored by testing protoplast isolation from ET under different maturation stages, ET pre-treatments such as drought or cold treatments, or the different compositions of CWD enzymes. Also, alternative CRISPR-Cas systems (e.g., Cas12a) could be tested for their protoplast delivery and genome editing efficiency [48]. The genetic transformation of *A. fraseri* and *P. taeda* primarily relies on biolistic and *Agrobacterium*-mediated methods, though efficiencies remain low compared to model plants. For *P. taeda*, the biolistic transformation of embryogenic callus or somatic embryos yields 1 to 5 stable transgenic events per 1000 bombarded embryos [49], while *Agrobacterium*-mediated transformation is less efficient, producing 0.1 to 1 stable integration events per 1000 infected embryos [49,50]. Similarly, *A. fraseri* transformation, though less studied, follows comparable protocols, with biolistic methods being the preferred approach due to the recalcitrance of conifers to *Agrobacterium* [51]. Both species face challenges such as low regeneration rates and difficulties in maintaining embryogenic cultures, which limit transformation efficiencies.

This protoplast system serves as a platform for functional gene characterization through protoplast delivery and transcriptional response analysis, enabling rapid gene function validation and the dissection of gene regulatory networks in conifers. It offers a pathway to advancing genetic studies on tree growth, development, and adaptation, with implications for enhancing forest resilience, sustainability, and productivity. CRISPR-RNP technology could facilitate improvements in stress tolerance, disease resistance, and biomass production in coniferous tree species. However, a major challenge remains in regenerating edited protoplasts into fully developed trees, which is a critical bottleneck that must be addressed to translate these genetic modifications into practical forestry applications [52,53]. Further research is essential to optimize regeneration protocols and unlock the full potential of CRISPR-based genome editing in conifers.

## 4. Methods

### 4.1. Pinus taeda Somatic Embryogenic Tissue Initiation and Maintenance

*P. taeda* juvenile cones were harvested in July 2021 by the Tree Improvement Program at NCSU, at the North Carolina Forest Service seed orchard, localized in Greensboro, NC. Cones were collected from 11 distinct *P. taeda* clones, transported within insulated containers with cold packs, and placed in a 4 °C cold chamber until dissection. Embryogenic tissue (ET) initiation was performed according to the protocol described by Pullman, 2018 [39], with some adaptations. Immature seeds were removed from each cone and disinfected with the incubation of 10% hydrogen peroxide solution for 15 min. After incubation, seeds were rinsed three times with sterile distilled water for 30 s and kept at 4 °C. For the dissection of the cleaned seeds, intact mega-gametophytes or zygotic embryos were removed, placed in PTIM medium (Table 2), and incubated at 26 °C in the dark for at least 60 days. The somatic embryogenic tissue proliferated from the dissected embryos was transferred to PTMM medium and maintained at 26 °C in dark. For the maintenance of the generated SE, young proliferated ET was harvested and placed in a new petri dish containing fresh PTMM (Table 2) medium every 10 days during incubation.

### 4.2. Abies fraseri Somatic Embryogenic Tissue Initiation and Maintenance

The *A. fraseri* somatic embryogenic (SE) tissue was acquired from the SE cryopreservation bank from the Christmas Tree Genetics Program at NC State. For the present work, the *A. fraseri* SE line 51-0025 was used. The ET was proliferated according to the protocol described by Pullman et al., 2016 [54], in which the young ET was transferred every 12 days into a fresh AFMM medium (Table 2).

### 4.3. Gene Amplification and Primer Design

#### 4.3.1. *Pinus taeda*

The established multi-omics integrative model for monolignol biosynthesis in *P. trichocarpa* [31,32,33,34] was used to extrapolate the best targeting gene families for genome editing in *P. taeda*. We simulated the impact of the entire gene families’ knockout in lignin content. The in silico gene perturbations were performed by specifying the model input as the target gene transcript abundances in the form of percentage expression of the wildtype level. The transcript abundances of the nine monolignol gene families were set to either 1%, the minimum transcript abundance used to train the model, (i.e., the entire gene family edited for loss-of-function) and 100% (i.e., wildtype level). A set of 19 gene expression profiles were simulated using the model to estimate changes in the corresponding protein abundance, steady-state metabolic fluxes, and lignin content in *P. taeda*.

The *PtPAL* gene CDS sequence was obtained from NCBI Genbank (Accession Number: U39792.1). Based on this sequence, a set of primers was designed to amplify 576 nt of the *PtPAL* 5′ end (Table 3). Genomic DNA from *P. taeda* was extracted from tree needles with the Quick-DNA Plant/Seed Miniprep Kit (Zymo Research, Irvine, CA, USA).

To confirm the precise genomic sequence for sgRNA design, the *PtPAL* gene was amplified with Q5 High-Fidelity DNA Polymerase (New England Biolabs, Ipswich, MA, USA), and the amplicons were cloned into the pMini 2.0 vector using the NEB^®^ PCR Cloning Kit (New England Biolabs). Bacterial colonies containing the recombinant plasmid were sequenced through Sanger sequencing (Genewiz, South Plainfield, NJ, USA). The sequencing reads were aligned to the *PtPAL* CDS (Accession Number: U39792.1) to find allelic variations.

#### 4.3.2. *Abies fraseri*

The *A. fraseri PDS* coding sequence (*AfPDS*) was obtained from a transcriptome assembly carried out by Dr. Jill Wegrzyn’s group at the University of Connecticut. RNA-seq reads were screened for contaminating sequences not of *A. fraseri* origin using Kraken2 [55], and trimmed for quality and adapter removal using Sickle 1.33 [56]. The clean trimmed reads were assembled using Trinity [57], and the assembled contigs annotated using the EnTAP pipeline [58]. In this annotation process, the sequence FraserFir9631 was identified as a probable ortholog of *15-cis-phytoene desaturase*.

We designed four sets of primers for the amplification of 215 bp from the 5′ region of the *AfPDS* gene (Table 3). *A. fraseri* genomic DNA extraction from the 51-0025 SE line, *AfPDS* gene amplification and cloning, and Sanger sequencing were performed as described for *P. taeda.*

### 4.4. Recombinant SpCas9 Expression and Purification

*Streptococcus pyogenes* Cas9 (SpCas9) recombinant protein was expressed in *Escherichia coli* Rosetta (DE3) strain (Novagen, Madison, WI, USA) harboring the pMJ915 plasmid [41]. Recombinant colonies were pre-inoculated in 3 mL of Luria–Bertani (LB) broth supplemented with 50 µg/mL of ampicillin for 16 h at 37 °C/250 rpm. After overnight culture, 1 mL of the pre-inoculum was inoculated in 1 L of LB broth supplemented with 100 µg/mL of ampicillin and incubated at 37 °C/250 rpm until the culture reached the log growth phase (OD600 of 0.4). Recombinant SpCas9 (rSpCas9) expression was induced by adding 0.25 mM of isopropylthio-β-galactoside (IPTG), and the culture was incubated for 16 h at 23 °C/250 rpm. The cell culture was centrifuged at 5000× *g* for 5 min, the supernatant was discarded, and the cell pellet was resuspended in 25 mL of 6×His Binding Buffer (NaH_2_PO_4_. 50 mM-pH 8.0, and NaCl 500 mM). Bacteria lysis was performed through sonication according to the following settings: 7 s ON, 25 s OFF, with a total time of 10 min at amplitude 20%. The cell lysate was centrifuged at 5000× *g* for 30 min at 4 °C, and the supernatant fraction that contained the rSpCas9 was collected.

For the purification of rSpCas9, 6 mL of Ni-NTA His Bind Resin (Millipore, Burlington, VT, USA) was pre-washed with 200 mL of 6×His Binding Buffer. Further, the supernatant fraction of the cell lysate was loaded into the column and washed with 6×His Wash Buffer (NaH_2_PO_4_. 50 mM at pH 8.0, NaCl 500 mM, and imidazole 20 mM). To elute the purified rSpCas9, 12 mL of 6xHis Elution Buffer (NaH_2_PO_4_. 50 mM at pH 8.0, NaCl 500 mM, and imidazole 250 mM) was loaded, and the flow-through was collected. To desalt the purified rSpCas9, the eluted flow-through was loaded into an Amicon Ultra-15 Centrifugal Filter Unit (cut-off, 100 kDa) (Millipore) and washed with 1×Phosphate Buffer Saline (PBS) (137 mM NaCl, 2.7 mM KCl, 4.3 mM Na_2_HPO_4_, 1.47 mM KH_2_PO_4_, at pH 7.4). The protein was quantified through a Bradford assay, and stored with 50% glycerol at −20 °C.

### 4.5. CRISPR sgRNA Design and CRISPR In Vitro Cleavage Assay

The sgRNA design to target *PtPAL* or *AfPDS* was performed using CRISPOR prediction tool (https://crispor.gi.ucsc.edu/, access date 10 March 2025) [59]. The sgRNAs were chosen according to their Moreno-Mateos efficiency scores, which better predicted the efficiency of cleavage of the sgRNAs transcribed in vitro [38]. The sgRNAs displaying the highest efficiency and outcome scores were chosen from the given list of candidates and functionally validated using in vitro cleavage assays [60]. Briefly, the DNA template for each sgRNA was generated by PCR containing 20 mM of each sgRNA primer, 20 mM BS6 primer, 1 µM T25_long primer, and 1 µM BS7 primer (Table 3). The DNA templates were then used for sgRNA synthesis by in vitro transcription using a HiScribe™ T7 Quick High Yield RNA Synthesis Kit (New England Biolabs). For the in vitro cleavage assay reactions, 7.8 µg of the rSpCas9 protein, 250 ng of linearized targeting plasmid (containing the sequences targeted by the correspondent sgRNAs), and 2 µL of the in vitro transcribed sgRNAs were incubated at 37 °C for 3 h in the reaction buffer (1 mM DDT, 5 mM MgCl_2_, 50 mM phosphate buffer at pH 7.5). DNA fragments from each reaction were purified with DNA Clean and Concentrator kit (Zymo Research). Reactions lacking the SpCas9, a sgRNA, or a non-specific sgRNA were used as negative controls. The reaction products were assessed by electrophoresis on a 1.5% agarose gel.

### 4.6. Protoplast Isolation

Protoplast isolation was performed according to Lin et al. (2014) [41], with modifications. The *P. taeda* ET was harvested and incubated in Cell-wall Digestion Solution 1 (CWDS1; Cellulase R10 1.5%, Macerozyme R10 0.4%, MES 20 mM at pH 5.7, KCl 20 mM, CaCl_2_ 10 mM, Mannitol 500 mM, and Bovine Serum Albumin 0.1%) or CWDS2 (CWDS1 supplemented with 0.5× PTMM media) following the proportion of 1:5 (wet tissue:CWDS volume). The digestion solution was incubated in a 150 mm petri dish for 3 or 16 h at room temperature in a rocking platform at 40 rpm. After incubation, the protoplasts were filtered through a sieve cup No. 35 (500 µM) (Sigma, St. Louis, USA). The petri-dish was washed with 5 mL of W5 solution (Glucose 5 mM, CaCl_2_ 125 mM, NaCl 154 mM, KCl 5 mM, and MES 2 mM at pH 5.7) diluted with 1× PTMM media (to a final concentration of 0.5× PTMM) to recover the remaining released protoplasts, and then filtered again through the sieve cup. The flow-through was re-filtered twice with 70 and 45 µm cell strainers, respectively. The second flow-through was centrifuged at 360× *g* for 5 min in a round bottom glass tube. After centrifugation, the supernatant was discarded, and the cell pellet was resuspended in 5 mL of MMG buffer (Mannitol 500 mM, MgCl_2_ 15 mM, and MES 4 mM at pH 5.7) diluted with 1× PTMM media (to a final concentration of 0.5× PTMM).

For *A. fraseri*, the ET was harvested and incubated in CWDS1 for 3 h at 26 °C/80 rpm. The released protoplasts were filtered twice through 70 and 45 µm cell strainers, respectively. The flow-through was centrifuged at 400× *g* for 5 min for cell pelleting. Protoplasts were washed with 5 mL of MMG buffer, centrifuged at 400× *g* for 5 min, and resuspended with 2 mL of MMG.

For protoplast purification, the 2 mL protoplast cell suspension was slowly pipetted on top of 10 mL of 0.6 M sucrose solution, and centrifuged at 400× *g* for 5 min. The protoplast cell layer (upper phase) was collected and mixed with 5 mL of either MMG or W5 buffer, and centrifuged at 400× *g* for 5 min. The supernatant was removed, and the cell pellet was resuspended with 1 mL of either MMG or W5 solution.

Protoplast samples were analyzed using an LSM 710 laser-scanning microscope (Zeiss, Oberkochen, Germany), and the protoplast concentration was assessed using a hemocytometer. The *P. taeda* protoplast viability was assessed using fluorescein diacetate (FDA) staining. After 16 h of incubation under CWDS, isolated protoplasts were mixed in a 1:1 ratio with a 0.1% FDA solution and incubated for 5 min at room temperature. Stained protoplasts were observed under UV light using a microscope, and viability was determined by calculating the proportion of fluorescent protoplasts out of a total count of 500. The *A. fraseri* protoplast viability was accessed immediately after protoplast transfection, following the same procedures described for the *P. taeda* protoplasts.

### 4.7. CRISPR-RNP Transfection

For RNP assembly, 90 µg of rSpCas9 and 30 µg of the sgRNAs were mixed in 1× NEB Buffer 3 and incubated for either 1 h at 37 °C (for *P. taeda*) or 20 min at 23 °C (*A. fraseri*) as described for other species [20,42]. CRISPR-RNPs (100 µL) were added to 1 mL of purified protoplasts (1 × 10^5^ cells), and then 1.1 mL of PEG-Ca^2+^ solution (PEG4000 40%, CaCl_2_ 100 mM, mannitol 200 mM) was added. The transfection reaction was incubated for 15 min at room temperature. The transfection reaction was washed with 4.4 mL of either WI (*A. fraseri*; Mannitol 500 mM, KCl 20 mM, and MES 4 mM) or W5 (*P. taeda*) solution, and the protoplasts were centrifuged at 400× *g* for 5 min. The supernatant was discarded, and protoplasts were washed again with 5 mL of WI (*A. fraseri*) or W5 (*P. taeda*) solution. The protoplasts were centrifuged again under the same conditions, and the supernatant was discarded. The cell pellets were resuspended in 1.5 mL of either regeneration media (*A. fraseri*; 1/2× MS, 1 mg/L NAA, 0.3 mg/L Kinetin, 5 mM MES, 0.5 M Mannitol) or W5 solution (*P. taeda*). The transfected protoplasts were incubated for 48 h (*A. fraseri*) or 72 h (*P. taeda*) in the dark at room temperature before DNA extraction (Figure S7). Protoplasts transfected with pUC19-*CaMV35S*-*gfp* and ddH20 were used as positive and negative transfection controls, respectively. The percentage of GFP-positive protoplasts over the total amount of protoplasts after 16 h provided a relative estimation for the transfection efficiency. The excitation and emission wavelengths used were 488 nm and 492 to 543 nm, respectively.

### 4.8. CRISPR-Driven Gene Editing Analysis

To detect CRISPR-driven mutations, the genomic DNA from each transfected protoplast sample was extracted with Quick-DNA Plant/Seed Miniprep Kit (Zymo Research). The 5′ regions of *PtPAL* and *AfPDS* were amplified with Q5 High-Fidelity DNA Polymerase (New England Biosciences). The *AfPDS* and *PtPAL* amplicons were purified with DNA Clean and Concentrator kit (Zymo Research), and sequenced by amplicon deep sequencing (Genewiz). The sequencing results were analyzed with Geneious Prime® 2025.0.3 software. INDEL edits occurring at the Cas9 cleavage sites were considered CRISPR-driven mutations. The CRISPR-editing efficiency was calculated by dividing the number of edited reads by the total number of sequencing reads mapped to the target regions.

## Figures and Tables

**Table 1:** In silico efficiency and outcome scores for CRISPR sgRNAs designed for *P. taeda* and *A. fraseri*.

			Efficiency Scores		Outcome Scores	
Name	Target Sequence	Position	Doench’16	Moreno-Mateos	Out-of-Frame	Lindel
PtPAL_gRNA1	CGAGCAGCCAAGGCCATGGA	106–125	55	57	65	84
PtPAL_gRNA2	CACTTGCGATCTTCGAGCAA	230–249	59	58	65	81
PtPAL_gRNA3	GAAACCAGTAGTGACACCAT	341–360	66	60	54	69
PtPAL_gRNA4	TGGGCATTTGCCAAGAACTC	434–453	51	66	80	77
PtPAL_gRNA5	CCCGGGCTGCCATGCTGGTT	479–498	39	72	62	68
AfPDS_gRNA1	TTGCCTTTGGACTCAGTGCA	61–80	55	50	53	91
AfPDS_gRNA2	TGCCTTTGGACTCAGTGCAA	62–81	59	59	60	77
AfPDS_gRNA3	GCAGCAGCAGGCTGGCATCT	110–129	45	49	63	86
AfPDS_gRNA4	TCTGGGAGGAGTGAATATTT	127–146	39	57	66	75

**Table 2:** Media components for SE initiation and maintenance for *P. taeda* and *A. fraseri*.

Media and Components (mg/L)			
	PTIM	PTMM	AFMM
NH_4_NO_3_	200	200	ˉ
KNO_3_	909.9	900	ˉ
KH_2_PO_4_	136.1	130	340
Ca(NO_3_)_2_·4H_2_O	236.2	230	ˉ
MgSO_4_·7H_2_O	246.5	250	394
Mg(NO_3_)_2_·6H_2_O	256.5	260	ˉ
MgCl_2_·6H_2_O	50	100	ˉ
CaSO_4_·2H_2_O	ˉ	ˉ	37.8
H_3_PO_4_	ˉ	ˉ	373
KI	4.15	4.15	0.083
H_3_BO_3_	15.5	15.5	2.48
MnSO_4_·H_2_O	10.5	10.5	18.6
ZnSO_4_·7H_2_O	14.668	14.4	5.76
NaMoO_4_·2H_2_O	0.125	0.125	0.103
CuSO_4_·5H_2_O	0.1725	0.125	3.75
COCl_2_·6H_2_O	0.125	0.125	0.012
NiCl_2_·6H_2_O	ˉ	ˉ	1.188
AgNO_3_	3.398	ˉ	ˉ
FeSO_4_·7H_2_O	13.9	13.9	ˉ
Na_2_EDTA	18.65	18.65	ˉ
Maltose	15,000	ˉ	ˉ
Sucrose	ˉ	15,000	10,000
Myo-inositol	100	500	1000
C_12_H_10_Mg_3_O_14_·9H_2_O	ˉ	ˉ	266
Casamino acids	500	ˉ	ˉ
L-glutamine	450	2500	2000
Thiamine HCl	1	1	1
Pyridoxine HCl	0.5	0.5	0.5
Nicotinic Acid	0.5	0.5	0.5
Glycine	2	2	ˉ
D-xylose	100	ˉ	ˉ
MES	250	ˉ	ˉ
Biotin	0.05	ˉ	ˉ
Folic acid	0.5	ˉ	ˉ
Vitamin B12	0.1	ˉ	ˉ
Vitamin E	0.1	ˉ	ˉ
α-ketoglutaric acid	100	ˉ	ˉ
Sodium thiosulfate	1 mM	ˉ	ˉ
NAA	2	ˉ	ˉ
2,4-D	ˉ	2	ˉ
BAP	0.63	0.333	1.1
Kinetin	0.61	ˉ	ˉ
Abscisic acid	ˉ	5	ˉ
24-epibrassinolide	2.1 μM	ˉ	ˉ
Gellan Gum	5000	5000	3000
pH	5.7	5.7	5.7

PTIM: *P. taeda* initiation media, PTMM: *P. taeda* maintenance media, AFMM: *A. fraseri* maintenance media.

**Table 3:** List of primers for *P. taeda* and *A. fraseri*.

Name	Sequence	Purpose
AfPDS_gRNA1	TAATACGACTCACTATAGTTGCCTTTGGACTCAGTGCAGTTTAAGAGCTATGCTGGAAACAGCATAGCAAGTTTAAATAAGG	In vitro transcription
AfPDS_gRNA2	TAATACGACTCACTATAGTGCCTTTGGACTCAGTGCAAGTTTAAGAGCTATGCTGGAAACAGCATAGCAAGTTTAAATAAGG	In vitro transcription
AfPDS_gRNA3	TAATACGACTCACTATAGGCAGCAGCAGGCTGGCATCTGTTTAAGAGCTATGCTGGAAACAGCATAGCAAGTTTAAATAAGG	In vitro transcription
AfPDS_gRNA4	TAATACGACTCACTATAGTCTGGGAGGAGTGAATATTTGTTTAAGAGCTATGCTGGAAACAGCATAGCAAGTTTAAATAAGG	In vitro transcription
AfPDS_Forward	GCGTTTCAAGGGTGGATTC	*AfPDS* amplification
AfPDS_Reverse	GTCCTTTGCAGGTTACATGC	*AfPDS* amplification
AfPDS_Forward_seq	ACACTCTTTCCCTACACGACGCTCTTCCGATCTGCGTTTCAAGGGTGGATTC	*AfPDS* NGS sequencing
AfPDS_Reverse_seq	GACTGGAGTTCAGACGTGTGCTCTTCCGATCTGCATGTAACCTGCAAAGGAC	*AfPDS* NGS sequencing
PtPAL_gRNA1	TAATACGACTCACTATAGCGAGCAGCCAAGGCCATGGAGTTTAAGAGCTATGCTGGAAACAGCATAGCAAGTTTAAATAAGG	In vitro transcription
PtPAL_gRNA2	TAATACGACTCACTATAGCACTTGCGATCTTCGAGCAAGTTTAAGAGCTATGCTGGAAACAGCATAGCAAGTTTAAATAAGG	In vitro transcription
PtPAL_gRNA3	TAATACGACTCACTATAGGAAACCAGTAGTGACACCATGTTTAAGAGCTATGCTGGAAACAGCATAGCAAGTTTAAATAAGG	In vitro transcription
PtPAL_gRNA4	TAATACGACTCACTATAGTGGGCATTTGCCAAGAACTCGTTTAAGAGCTATGCTGGAAACAGCATAGCAAGTTTAAATAAGG	In vitro transcription
PtPAL_gRNA5	TAATACGACTCACTATAGCCCGGGCTGCCATGCTGGTTGTTTAAGAGCTATGCTGGAAACAGCATAGCAAGTTTAAATAAGG	In vitro transcription
PtPAL_Forward	CAGCAGCAGAAATAACGCA	*PtPAL* amplification
PtPAL_Reverse	CTGTTGAATGCGTGGCTGA	*PtPAL* amplification
PtPAL_Forward_seq	ACACTCTTTCCCTACACGACGCTCTTCCGATCTCAGCAGCAGAAATAACGCA	*PtPAL* NGS sequencing
PtPAL_Reverse_seq	GACTGGAGTTCAGACGTGTGCTCTTCCGATCTCTGTTGAATGCGTGGCTGA	*PtPAL* NGS sequencing
BS6	AAAAAAAGCACCGACTCGGTGCCACTTTTTCAAGTTGATAACGGACTAGCCTTATTTAAACTTGCTATGCTGTTTCCAGC	In vitro transcription
BS7	AAAAAAAGCACCGACTCGGTGC	In vitro transcription
T25-long	GAAATTAATACGACTCACTATAG	In vitro transcription

## Data Availability

Data are contained within the article and the Supplementary Materials.

## Supplementary Materials

The following supporting information can be downloaded at: https://www.mdpi.com/article/10.3390/plants14070996/s1, Figure S1: Screening of monolignol target gene families for improved wood properties; Figure S2: Identification *PtPAL* gene variants in *P. taeda* and CRISPR sgRNA design; Figure S3: CRISPR-Cas9 in vitro cleavage assays; Figure S4: Protoplasts post-transfection recovery in MMG and PTMM medium; Figure S5: CRISPR-mediated *AfPDS* knockout through RNP transfection of SE-originated protoplasts; Figure S6: Assessment of protoplast viability in *P. taeda*; Figure S7: Schematic representation of step procedures for ET-originated protoplast CRISPR-RNP delivery; Figure S8: Assessment of protoplast viability in *A. fraseri*.

## References

[1] De Frenne P., Lenoir J., Luoto M., Scheffers B.R., Zellweger F., Aalto J., Ashcroft M.B., Christiansen D.M., Decocq G., De Pauw K. (2021). Forest microclimates and climate change: Importance, drivers and future research agenda. Glob. Change Biol..

[2] Food and Agriculture Organization of the United Nations (2020). Global Forest Resources Assessment. https://www.fao.org/interactive/forest-resources-assessment/2020/en/.

[3] Xiao S., Chen C., Xia Q., Liu Y., Yao Y., Chen Q., Hartsfield M., Brozena A., Tu K., Eichhorn S.J. (2021). Lightweight, strong, moldable wood via cell wall engineering as a sustainable structural material. Science.

[4] U.S. Environmental Protection Agency (2021). Greenhouse Gas Reporting Program (GHGRP). https://www.epa.gov/ghgreporting/ghgrp-pulp-and-paper#map-facilities.

[5] Vanholme R., Cesarino I., Rataj K., Xiao Y., Sundin L., Goeminne G., Kim H., Cross J., Morreel K., Araujo P. (2013). Caffeoyl shikimate esterase (CSE) is an enzyme in the lignin biosynthetic pathway in Arabidopsis. Science.

[6] Sulis D.B., Jiang X., Yang C., Marques B.M., Matthews M.L., Miller Z., Lan K., Cofre-Vega C., Liu B., Sun R. (2023). Multiplex CRISPR editing of wood for sustainable fiber production. Science.

[7] Sulis D.B., Wang J.P. (2020). Regulation of lignin biosynthesis by post-translational protein modifications. Front. Plant Sci..

[8] Susaeta A., Carter D.R., Adams D.C. (2014). Impacts of climate change on economics of forestry and adaptation strategies in the southern United States. J. Agric. Appl. Econ..

[9] Pureswaran D.S., Roques A., Battisti A. (2018). Forest insects and climate change. Curr. For. Rep..

[10] United States Department of Agriculture, National Agricultural Statistics Service (2017). Census of Agriculture. www.nass.usda.gov/AgCensus.

[11] Potter K.M., Hargrove W.W., Koch F.H. Predicting climate change extirpation risk for central and southern Appalachian forest tree species. Proceedings of the Conference on the Ecology and Management of High-Elevation Forests in the Central and Southern Appalachian Mountains.

[12] Kaylor S.D., Hughes M.J., Franklin J.A. (2017). Recovery trends and predictions of Fraser fir (*Abies fraseri*) dynamics in the Southern Appalachian Mountains. Can. J. For. Res..

[13] Mckeever K.M., Chastagner G.A., Pathology P., State W. (2016). A Survey of *Phytophthora* spp. Associated with Abies in U.S. Christmas Tree Farms. Plant Disease.

[14] Prunier J., Verta J.P., MacKay J.J. (2016). Conifer genomics and adaptation: At the crossroads of genetic diversity and genome function. New Phytol..

[15] Kovach A., Wegrzyn J.L., Parra G., Holt C., Bruening G.E., Loopstra C.A., Hartigan J., Yandell M., Langley C.H., Korf I. (2010). The *Pinus taeda* genome is characterized by diverse and highly diverged repetitive sequences. BMC Genom..

[16] Liu B., Liu J., Yu J., Wang Z., Sun Y., Li S., Lin Y.-C.J., Chiang V.L., Li W., Wang J.P. (2021). Transcriptional reprogramming of xylem cell wall biosynthesis in tension wood. Plant Physiol..

[17] Chen H., Wang J.P., Liu H., Li H., Lin Y.-C.J., Shi R., Yang C., Gao J., Zhou C., Li Q. (2019). Hierarchical transcription factor and chromatin binding network for wood formation in *Populus trichocarpa*. Plant Cell.

[18] Wang Z., Mao Y., Guo Y., Gao J., Liu X., Li S., Lin Y.-C.J., Chen H., Wang J.P., Chiang V.L. (2020). MYB transcription factor161 mediates feedback regulation of secondary wall-associated NAC-Domain1 family genes for wood formation. Plant Physiol..

[19] González M.N., Massa G.A., Andersson M., Turesson H., Olsson N., Fält A.-S., Storani L., Décima Oneto C.A., Hofvander P., Feingold S.E. (2020). Reduced enzymatic browning in potato tubers by specific editing of a polyphenol oxidase gene via ribonucleoprotein complexes delivery of the CRISPR/Cas9 system. Front. Plant Sci..

[20] Woo J.W., Kim J., Kwon S.I., Corvalán C., Cho S.W., Kim H., Kim S.-G., Kim S.-T., Choe S., Kim J.-S. (2015). DNA-free genome editing in plants with preassembled CRISPR-Cas9 ribonucleoproteins. Nat. Biotechnol..

[21] Fan Y., Xin S., Dai X., Yang X., Huang H., Hua Y. (2020). Efficient genome editing of rubber tree (*Hevea brasiliensis*) protoplasts using CRISPR/Cas9 ribonucleoproteins. Ind. Crops Prod..

[22] Pavese V., Moglia A., Abbà S., Milani A.M., Torello Marinoni D., Corredoira E., Martínez M.T., Botta R. (2022). First Report on Genome Editing via Ribonucleoprotein (RNP) in Castanea sativa Mill. Int. J. Mol. Sci..

[23] Qin G., Gu H., Ma L., Peng Y., Deng X.W., Chen Z., Qu L.-J. (2007). Disruption of phytoene desaturase gene results in albino and dwarf phenotypes in *Arabidopsis* by impairing chlorophyll, carotenoid, and gibberellin biosynthesis. Cell Res..

[24] Murovec J., Guček K., Bohanec B., Avbelj M., Jerala R. (2018). DNA-free genome editing of Brassica oleracea and *B. rapa* protoplasts using CRISPR-Cas9 ribonucleoprotein complexes. Front. Plant Sci..

[25] Banakar R., Schubert M., Collingwood M., Vakulskas C., Eggenberger A.L., Wang K. (2020). Comparison of CRISPR-Cas9/Cas12a ribonucleoprotein complexes for genome editing efficiency in the rice phytoene desaturase (*OsPDS*) gene. Rice.

[26] Wu S., Zhu H., Liu J., Yang Q., Shao X., Bi F., Hu C., Huo H., Chen K., Yi G. (2020). Establishment of a PEG-mediated protoplast transformation system based on DNA and CRISPR/Cas9 ribonucleoprotein complexes for banana. BMC Plant Biol..

[27] Ma M., Zhang C., Yu L., Yang J., Li C. (2024). CRISPR/Cas9 ribonucleoprotein mediated DNA-free genome editing in larch. For. Res..

[28] Poovaiah C., Phillips L., Geddes B., Reeves C., Sorieul M., Thorlby G. (2021). Genome editing with CRISPR/Cas9 in *Pinus radiata* (D. Don). BMC Plant Biol..

[29] Cao H.X., Vu G.T.H., Gailing O. (2022). From Genome Sequencing to CRISPR-Based Genome Editing for Climate-Resilient Forest Trees. Int. J. Mol. Sci..

[30] Chang S., Mahon E.L., MacKay H.A., Rottmann W.H., Strauss S.H., Pijut P.M., Powell W.A., Coffey V., Lu H., Mansfield S.D. (2018). Genetic engineering of trees: Progress and new horizons. Vitr. Cell. Dev. Biol.-Plant.

[31] Wang J.P., Matthews M.L., Williams C.M., Shi R., Yang C., Tunlaya-Anukit S., Chen H.-C., Li Q., Liu J., Lin C.-Y. (2018). Improving wood properties for wood utilization through multi-omics integration in lignin biosynthesis. Nat. Commun..

[32] Wang J.P., Liu B., Sun Y., Chiang V.L., Sederoff R.R. (2019). Enzyme-enzyme interactions in monolignol biosynthesis. Front. Plant Sci..

[33] Matthews M.L., Wang J.P., Sederoff R., Chiang V.L., Williams C.M. (2020). Modeling cross-regulatory influences on monolignol transcripts and proteins under single and combinatorial gene knockdowns in *Populus trichocarpa*. PLoS Comput. Biol..

[34] Matthews M.L., Wang J.P., Sederoff R., Chiang V.L., Williams C.M. (2021). A multiscale model of lignin biosynthesis for predicting bioenergy traits in *Populus trichocarpa*. Comput. Struct. Biotechnol. J..

[35] Whetten R., Sederoff R. (1995). Lignin biosynthesis. Plant Cell.

[36] Shi R., Shuford C.M., Wang J.P., Sun Y.-H., Yang Z., Chen H.-C., Tunlaya-Anukit S., Li Q., Liu J., Muddiman D.C. (2013). Regulation of phenylalanine ammonia-lyase (PAL) gene family in wood forming tissue of *Populus trichocarpa*. Planta.

[37] Zhang X., Liu C.-J. (2015). Multifaceted regulations of gateway enzyme phenylalanine ammonia-lyase in the biosynthesis of phenylpropanoids. Mol. Plant.

[38] Vejnar C.E., Moreno-Mateos M.A., Cifuentes D., Bazzini A.A., Giraldez A.J. (2016). Optimized CRISPR–Cas9 system for genome editing in zebrafish. Cold Spring Harb. Protoc..

[39] Pullman G.S. (2018). Embryogenic tissue initiation in loblolly pine (*Pinus taeda* L.). Step Wise Protocols for Somatic Embryogenesis of Important Woody Plants.

[40] Cairney J., Pullman G.S. (2007). The cellular and molecular biology of conifer embryogenesis. New Phytol..

[41] Lin Y.-C., Li W., Chen H., Li Q., Sun Y.-H., Shi R., Lin C.-Y., Wang J.P., Chen H.-C., Chuang L. (2014). A simple improved-throughput xylem protoplast system for studying wood formation. Nat. Protoc..

[42] Hsu C.-T., Lee W.-C., Cheng Y.-J., Yuan Y.-H., Wu F.-H., Lin C.-S. (2021). Genome editing and protoplast regeneration to study plant–pathogen interactions in the model plant *Nicotiana benthamiana*. Front. Genome Ed..

[43] Haas B.J., Papanicolaou A., Yassour M., Grabherr M., Blood P.D., Bowden J., Couger M.B., Eccles D., Li B., Lieber M. (2013). De novo transcript sequence reconstruction from RNA-seq using the Trinity platform for reference generation and analysis. Nat. Protoc..

[44] Chou E.Y. (2016). Understanding the Patterned Deposition of Lignin in Secondary Cell Walls.

[45] Masani M.Y.A., Noll G.A., Parveez G.K.A., Sambanthamurthi R., Prüfer D. (2014). Efficient transformation of oil palm protoplasts by PEG-mediated transfection and DNA microinjection. PLoS ONE.

[46] Malnoy M., Viola R., Jung M.-H., Koo O.-J., Kim S., Kim J.-S., Velasco R., Nagamangala Kanchiswamy C. (2016). DNA-free genetically edited grapevine and apple protoplast using CRISPR/Cas9 ribonucleoproteins. Front. Plant Sci..

[47] Carlsen F.M., Johansen I.E., Yang Z., Liu Y., Westberg I.N., Kieu N.P., Jørgensen B., Lenman M., Andreasson E., Nielsen K.L. (2022). Strategies for efficient gene editing in protoplasts of *Solanum tuberosum* theme: Determining gRNA efficiency design by utilizing protoplast. Front. Genome Ed..

[48] Zhang Y., Ren Q., Tang X., Liu S., Malzahn A.A., Zhou J., Wang J., Yin D., Pan C., Yuan M. (2021). Expanding the scope of plant genome engineering with Cas12a orthologs and highly multiplexable editing systems. Nat. Commun..

[49] Tang W., Newton R. (2003). Genetic transformation of conifers and its application in forest biotechnology. Plant Cell Rep..

[50] Klimaszewska K., Hargreaves C., Lelu-Walter M.-A., Trontin J.-F. (2016). Advances in conifer somatic embryogenesis since year 2000. Vitr. Embryog. High. Plants.

[51] Trontin J.-F., Walter C., Klimaszewska K., Park Y.-S., Lelu-Walter M.-A. (2007). Recent progress in genetic transformation of four *Pinus* spp. Transgenic Plant J..

[52] Bewg W.P., Ci D., Tsai C.-J. (2018). Genome editing in trees: From multiple repair pathways to long-term stability. Front. Plant Sci..

[53] Tsai C.-J., Xue L.-J. (2015). CRISPRing into the woods. GM Crops Food.

[54] Pullman G.S., Olson K., Egertsdotter U., Bucalo K. (2016). Fraser fir somatic embryogenesis: High frequency initiation, maintenance, embryo development, germination and cryopreservation. New For..

[55] Wood D.E., Lu J., Langmead B. (2019). Improved metagenomic analysis with Kraken 2. Genome Biol..

[56] Joshi N., Fass J. (2014). Sickle: A Sliding-Window, Adaptive, Quality-Based Trimming Tool for FastQ Files.

[57] Grabherr M.G., Haas B.J., Yassour M., Levin J.Z., Thompson D.A., Amit I., Adiconis X., Fan L., Raychowdhury R., Zeng Q. (2011). Trinity: Reconstructing a full-length transcriptome without a genome from RNA-Seq data. Nat. Biotechnol..

[58] Hart A.J., Ginzburg S., Xu M., Fisher C.R., Rahmatpour N., Mitton J.B., Paul R., Wegrzyn J.L. (2020). EnTAP: Bringing faster and smarter functional annotation to non-model eukaryotic transcriptomes. Mol. Ecol. Resour..

[59] Concordet J.-P., Haeussler M. (2018). CRISPOR: Intuitive guide selection for CRISPR/Cas9 genome editing experiments and screens. Nucleic Acids Res..

[60] Pattanayak V., Lin S., Guilinger J.P., Ma E., Doudna J.A., Liu D.R. (2013). High-throughput profiling of off-target DNA cleavage reveals RNA-programmed Cas9 nuclease specificity. Nat. Biotechnol..

