# Supplementary material for: A Protoplast System for CRISPR-Cas Ribonucleoprotein Delivery in Pinus taeda and Abies fraseri
Source: Plants (Basel). 2025 Mar 22;14(7):996. doi: 10.3390/plants14070996 (PMC11990275; doi:10.3390/plants14070996)
Supplement: Supplementary file 1 [file plants-14-00996-s001.zip › plants-3509328-supplementary.pdf]

SUPPLEMENTARY FIGURES

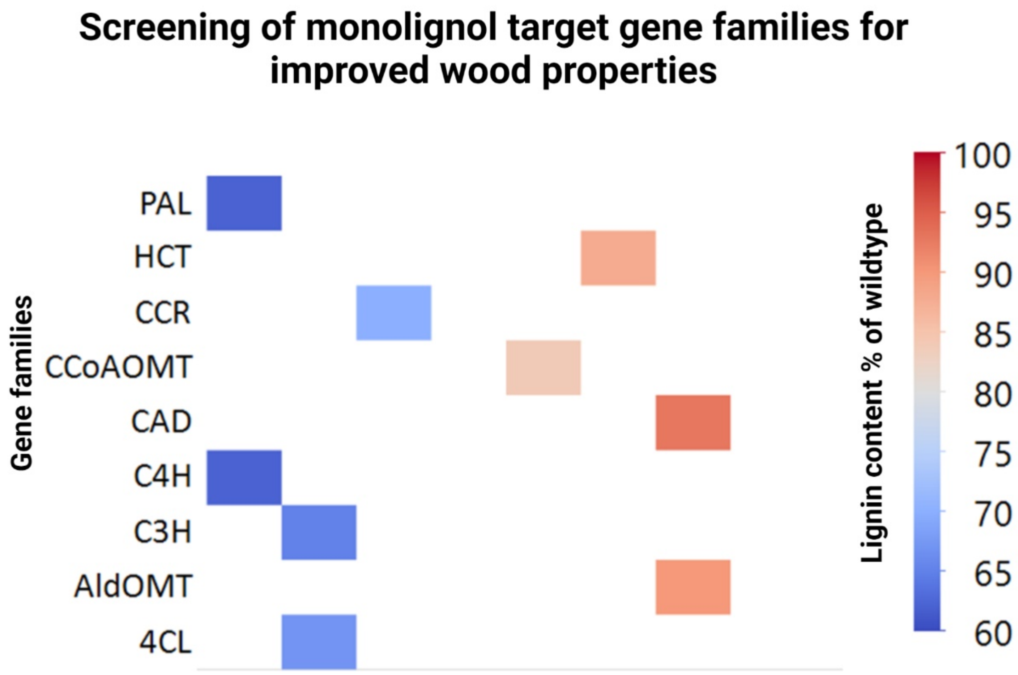

Figure S1. Screening of monolignol target gene families for improved wood properties.

We assessed the effect of CRISPR-based knockout targeting on monolignol gene families and evaluated their impact on lignin content using our established predictive model for monolignol biosynthesis (Wang et al. 2018; Wang et al. 2019; Matthews et al. 2020, 2021). This screening aimed to identify gene families that can potentially improve wood properties.

(A)

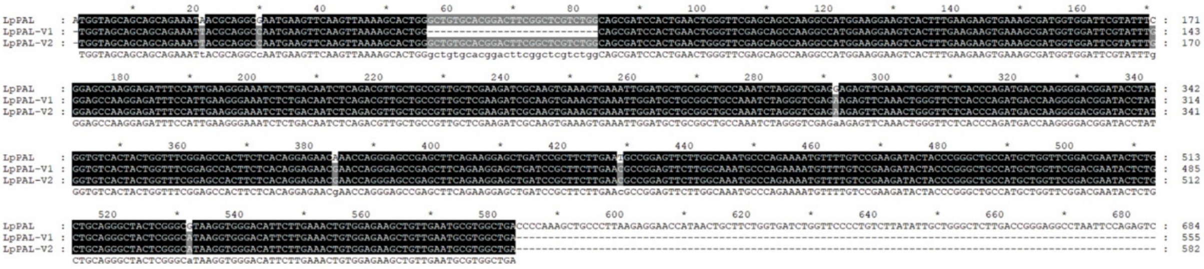

(B)

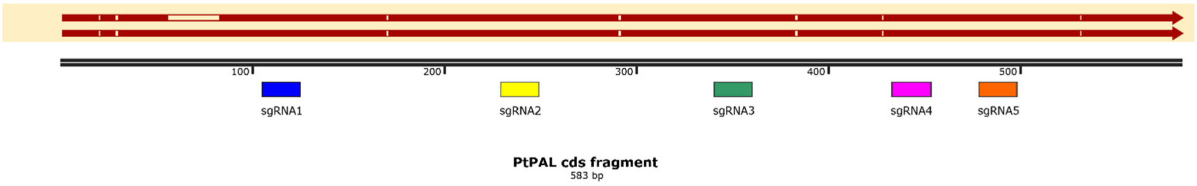

Figure S2. Identification *PtPAL* gene variants in *P. taeda* and CRISPR sgRNA design.

The 583 bp amplicon of *PtPAL* gene was sequenced through Sanger. (A) Sequencing results were aligned with ClustalW with the *PtPAL* sequence deposited on Genebank (Accession Number: U39792.1). Sequences highlighted in black are completely aligned, in gray partially aligned, and in white are not aligned. (B) Schematic representation of the *PtPAL* 583 bp amplicon, aligned with the two identified variants in (red), where SNPs and deletions are represented under the faint positions. sgRNA positions are represented in the squares colored by blue (sgRNA1), yellow (sgRNA2), green (sgRNA3), pink (sgRNA4) and orange (sgRNA5).

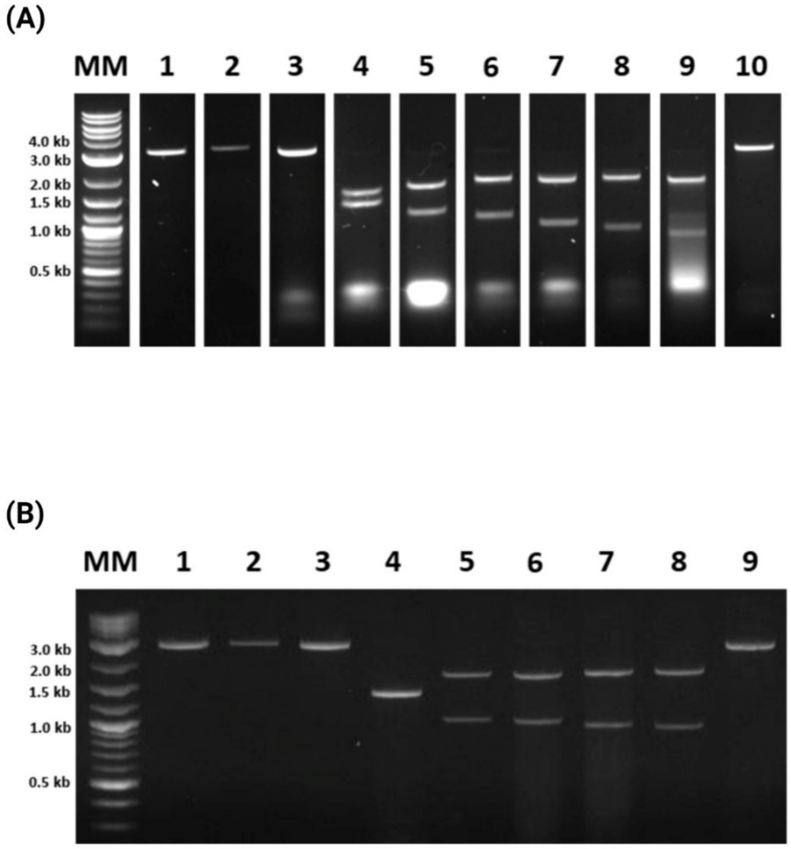

Figure S3. CRISPR-Cas9 in vitro cleavage assays.

(A) Electrophoretic analysis of the cleavage assay reactions targeting *PtPAL* after 3 h incubation, where (MM) represents molecular marker; (1) negative control without Cas9 and gRNA; (2) negative control without gRNA; (3) negative control without Cas9; (4) positive control reaction with Amp_gRNA; (5) gRNA1 reaction; (6) gRNA2 reaction; (7) gRNA3 reaction; (8) gRNA4 reaction; (9) gRNA5 reaction; (10) negative control with a scramble gRNA. (B) Electrophoretic analysis of the cleavage assay reactions targeting *AfPDS* after 3 h incubation, where (MM) represents molecular marker; (1) negative control without Cas9 and gRNA; (2) negative control without gRNA; (3) negative control without Cas9; (4) positive control reaction with Amp_gRNA; (5) gRNA1 reaction; (6) gRNA2 reaction; (7) gRNA3 reaction; (8) gRNA4 reaction; (9) negative control with a scramble gRNA.

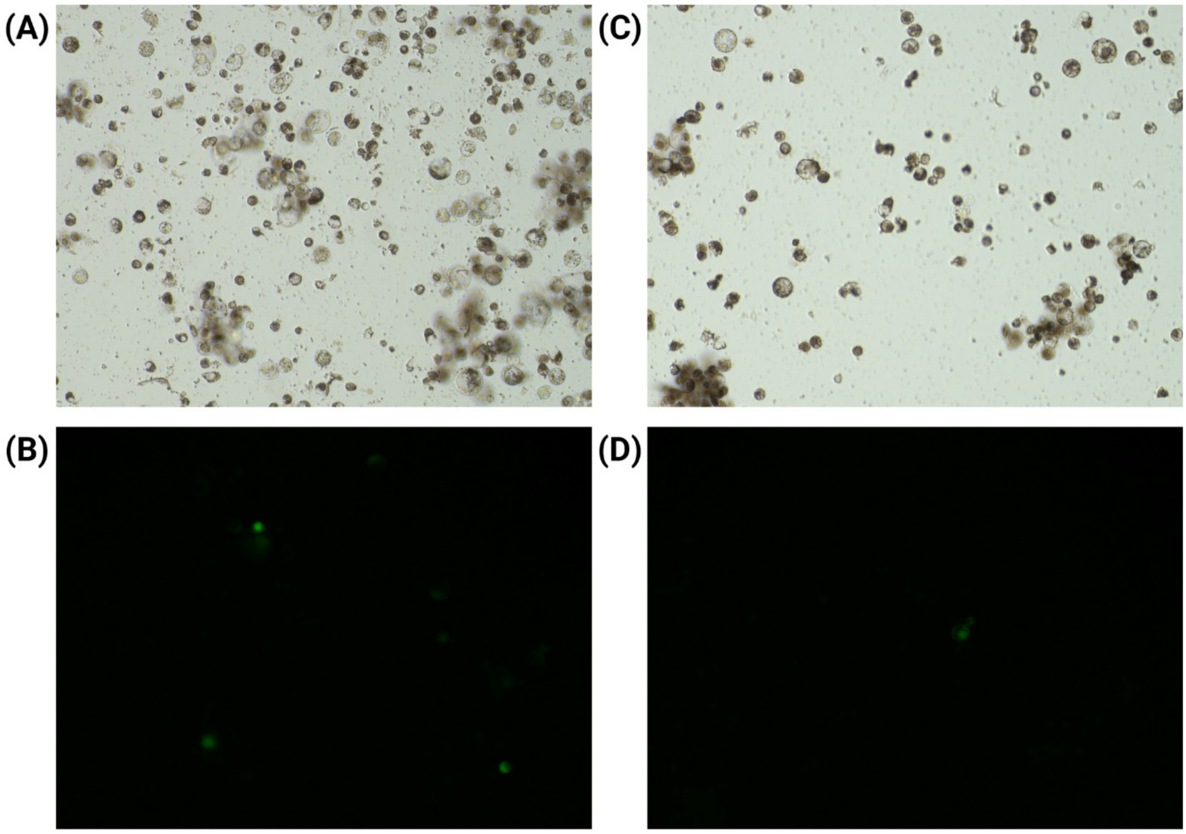

Figure S4. Protoplasts post-transfection recovery in MMG and PTMM medium.

P. taeda ET-isolated protoplasts of 21-063 line were transfected with a reporter plasmid, and incubated for 16 hours in MMG (A and B), PTMM medium (C and D). Protoplasts were observed in bright field (A and C), and in UV light (B and D) for assessment of transfection efficiencies.

(A) (B)

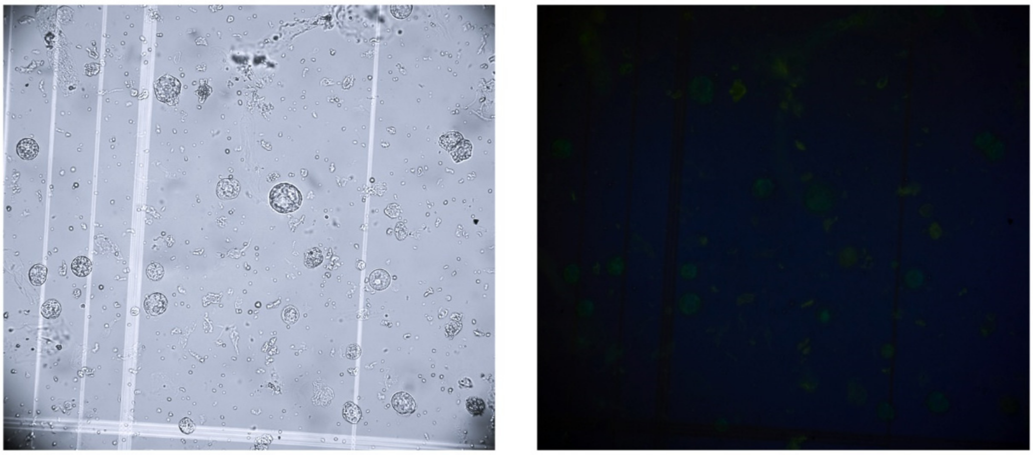

(C)

		gRNA4 targeting site																				PAM			
Reference	5'	T	C	T	G	G	G	A	G	G	A	G	T	G	A	A	T	A	T	T	T	C	G	G	3'	%
	5'	T	C	T	G	G	G	A	G	G	A	G	T	G	A	-	-	-	-	T	T	C	G	G	3'	0.02
	5'	T	C	T	G	G	G	A	G	G	A	G	T	G	A	A	T	A	-	T	T	C	G	G	3'	0.01
	5'	T	C	T	G	G	G	A	G	G	A	G	T	G	A	A	-	-	T	T	T	C	G	G	3'	0.01
	5'	T	C	T	G	G	G	A	G	G	A	G	T	G	A	-	T	A	T	T	T	C	G	G	3'	0.005
	5'	T	C	T	G	G	G	A	G	G	A	G	T	G	A	A	T	-	T	T	T	C	G	G	3'	0.005
	5'	T	C	T	G	G	G	A	G	G	A	G	T	G	A	A	-	-	A	T	T	C	G	G	3'	0.005

Figure S5. CRISPR-mediated *AfPDS* knockout through RNP transfection of ET-originated protoplasts.

A. fraseri protoplasts of 51-0025 line were isolated, and transfected with CRISPR-RNP complexes targeting different regions of *AfPDS* gene sequence. Additionally, protoplasts were transfected with pUC19-GFP reporter plasmid. After 16 hours of transfection, protoplasts were observed under bright field (A), and UV light (B), for assessment of transfection efficiency. Samples transfected with CRISPR-RNPs were analyzed through amplicon deep sequencing. CRISPR-associated *AfPDS* variants were observed for samples transfected with sgRNA4 (C), where basepairs highlighted in gray represent the PAM sites, in red deletions, and green base-exchanges.

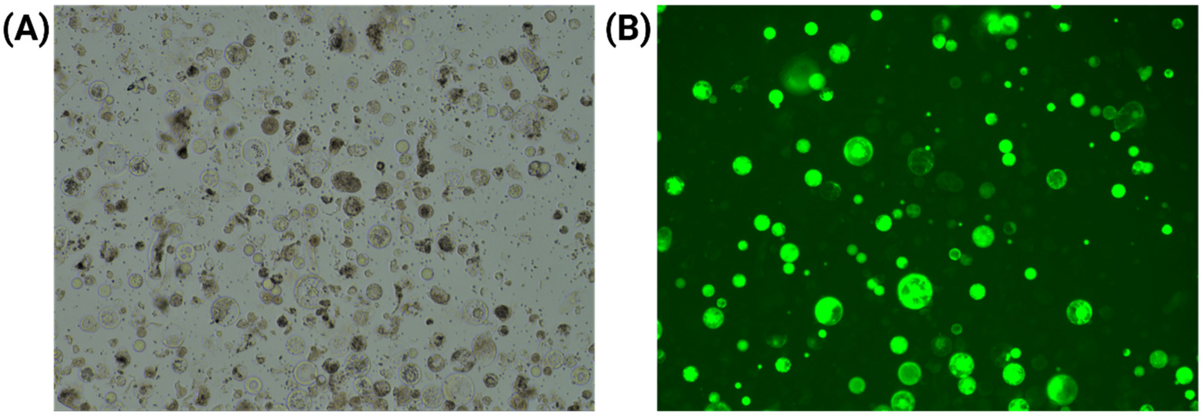

Figure S6. Assessment of protoplast viability in *P. taeda*.

After 16 hours of incubation for cell-wall digestion, protoplasts isolated from P. taeda ET masses were stained with FDA and observed under UV light.

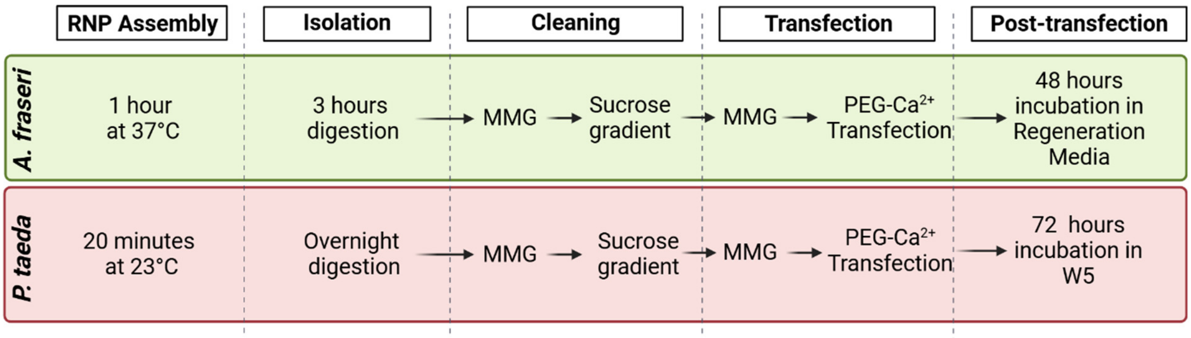

Figure S7. Schematic representation of step procedures for ET-originated protoplast CRISPR-RNP delivery.

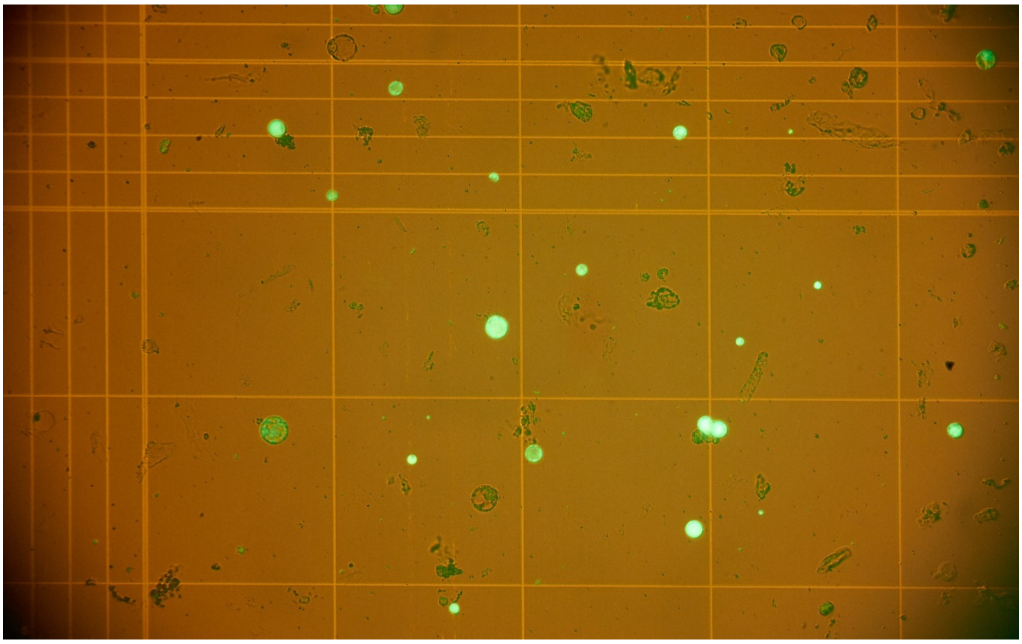

Figure S8. Assessment of protoplast viability in *A. fraseri*.

Immediately after PEG-Ca^{2+} transfection, protoplasts from *A. fraseri* ET masses were stained with FDA and observed under UV light.
